# Effect of Plant-Based Proteins on Recovery from Resistance Exercise-Induced Muscle Damage in Healthy Young Adults—A Systematic Review

**DOI:** 10.3390/nu17152571

**Published:** 2025-08-07

**Authors:** Karuppasamy Govindasamy, Koulla Parpa, Borko Katanic, Cain C. T. Clark, Masilamani Elayaraja, Ibnu Noufal Kambitta Valappil, Corina Dulceanu, Vlad Adrian Geantă, Gloria Alexandra Tolan, Hassane Zouhal

**Affiliations:** 1 Department of Sports, Recreation and Wellness, Symbiosis International (Deemed University), Hyderabad Campus, Modallaguda (V), Nandigama (M), Rangareddy, Hyderabad 509217, India; 2 Faculty of Sport and Exercise Science, UCLan University of Cyprus, Pyla 7080, Cyprus; kparpa@uclan.ac.uk; 3 Montenegrin Sports Academy, 81000 Podgorica, Montenegro; borkokatanic@gmail.com; 4 College of Life Sciences, Birmingham City University, Birmingham B15 3TN, UK; cain.clark@bcu.ac.uk; 5 Department of Physical Education and Sports, Pondicherry University, Puducherry 605014, India; elaya.cricket@gmail.com (M.E.); noufalibnukv70@gmail.com (I.N.K.V.); 6 Faculty of Physical Education and Sport, Aurel Vlaicu University of Arad, 310330 Arad, Romania; corina.dulceanu@yahoo.com; 7 Doctoral School of Sport Science and Physical Education, Pitești University Center, National University of Science and Technology Politehnica Bucharest, 110253 Pitești, Romania; 8 Multidisciplinary Doctoral School, “Vasile Goldis” Western University, 310419 Arad, Romania; gloria.tolan@yahoo.de; 9 Institut International des Sciences du Sport (2I2S), 35850 Irodouer, France; 10 Laboratoire Optimisation de la Performance Sportive (LR09SEP01), Centre National de la Médecine et des Sciences des Sports, Tunis 1004, Tunisia

**Keywords:** soy, hemp, plant-based protein, athletic performance, recovery, strength training

## Abstract

Background: Plant-based protein supplementation in supporting muscle recovery following resistance exercise remains an area of growing interest, particularly among vegan athletes, as a potential alternative to animal-based proteins. This systematic review aimed to evaluate the effectiveness of plant-based proteins on recovery from resistance exercise-induced muscle damage in healthy young adults. Methods: A systematic and comprehensive search was administered in eight databases up to 1 May 2025, identifying 1407 articles. Following deduplication and screening, 24 studies met the eligibility criteria, including 22 randomized controlled trials and 2 non-randomized studies, with the majority from high income western countries. Results: Interventions primarily involved soy, pea, rice, hemp, potato, and blended plant protein sources, with doses ranging from 15 to 50 g, typically administered post resistance exercise. Outcomes assessed included muscle protein synthesis (MPS), delayed-onset muscle soreness (DOMS), inflammatory biomarkers, muscle function, and fatigue. The review findings reaffirm that single-source plant proteins generally offer limited benefits compared to animal proteins such as whey, particularly in acute recovery settings, a limitation well-documented consistently in the literature. However, our synthesis highlights that well-formulated plant protein blends (e.g., combinations of pea, rice, and canola) can stimulate MPS at levels comparable to whey when consumed at adequate doses (≥30 g with ~2.5 g leucine). Some studies also reported improvements in subjective recovery outcomes and reductions in muscle damage biomarkers with soy or pea protein. However, overall evidence remains limited by small sample sizes, moderate to high risk of bias, and heterogeneity in intervention protocols, protein formulations, and outcome measures. Risk of bias assessments revealed concerns related to detection and reporting bias in nearly half the studies. Due to clinical and methodological variability, a meta-analysis was not conducted. Conclusion: plant-based proteins particularly in the form of protein blends and when dosed appropriately, may support muscle recovery in resistance-trained individuals and offer a viable alternative to animal-based proteins. However, further high-quality, long-term trials in vegan populations are needed to establish definitive recommendations for plant protein use in sports nutrition.

## 1. Introduction

Plant-based proteins are speculated to offer potential health benefits including cardiometabolic disease risk reduction and blood glucose regulation [1]. For past decades, there is a growing interest on exploring plant-based proteins for improving the athletic performance and recovery [2]. Athletic dietary landscape is witnessing a significant shift towards plant-based eating, driven by a confluence of ethical, environmental, and health-related considerations [3]. This trend has notably increased interest in plant-based nutrition among athletic populations, including individuals engaged in regular resistance training [4]. Modeling studies suggest that adding larger amount of plant-based proteins to a routine athletic meal plan, can meet leucine and total protein requirements, potentially achieving levels comparable to those provided by typical servings of animal proteins [5]. While acute studies demonstrated that animal proteins induce greater muscle protein synthesis (MPS), chronic studies showed that plant-based proteins can yield similar adaptations if consumed in adequate amounts. Resistance exercise, a fundamental component of athletic development and general fitness, characteristically induces muscle damage, most notably through eccentric contractions [6,7]. However, it is also well-established that strenuous or unaccustomed, regardless of contraction type, cause microtears and inflammation, eliciting delayed-onset muscle soreness (DOMS) and making it a relevant model for studying recovery in both eccentric and non-eccentric resistance exercise contexts [8].

Adequate protein intake is widely recognized as critical for facilitating muscle repair and promoting recovery processes [9,10]. Traditionally, animal-derived proteins, such as whey, have been favored due to their rich profiles of essential amino acids, especially leucine, which plays a key role in stimulating MPS [11]. However, vegan athletes, who consciously avoid all animal products, must rely exclusively on plant-based protein sources [12]. These sources often present different amino acid profiles, sometimes with lower leucine content compared to their animal-based counterparts [13]. This disparity raises pertinent questions regarding the efficacy of plant-based proteins in supporting optimal recovery from exercise-induced muscle damage, particularly for the growing number of young adults adopting vegan diets while concurrently pursuing resistance training for health and performance enhancement [4,14].

Despite the escalating popularity of plant-based diets, the scientific literature examining the specific effects of plant-based proteins on muscle recovery following resistance exercise remains somewhat fragmented [2,4]. While some research suggests that appropriately dosed plant-based protein blends can rival animal proteins in stimulating MPS [13], other studies highlight potential challenges such as lower bioavailability or incomplete amino acid profiles in certain plant sources [15]. This prevailing uncertainty is especially relevant for vegan athletes who may face challenges in meeting their protein requirements without animal-derived sources, potentially impacting their recovery and athletic performance. Therefore, a systematic review is warranted to comprehensively map the existing evidence, identify knowledge gaps, and clarify whether plant-based proteins can effectively support recovery from resistance exercise-induced muscle damage in healthy young adults. By synthesizing available data on intervention protocols, outcome measures, and study designs, this review aims to build a foundation for evidence-based nutritional recommendations, particularly for vegan athletes, and to guide future research in this potentially important and interesting area. The primary aim of this systematic review is to examine the effect of plant-based protein supplementation on recovery from resistance exercise-induced muscle damage in healthy young adults, with a particular focus on understanding the implications for vegan athletes who depend solely on plant-based sources for their protein needs.

## 2. Materials and Methods

This systematic review was conducted and reported in adherence to the PRISMA (Preferred Reporting Items for Systematic Reviews and Meta-Analyses) guidelines, utilizing the PERSiST (Preferred Reporting Items for Systematic Reviews in Sport and Exercise Science) guidance to ensure transparent and comprehensive reporting of methods and findings [16]. The checklist is provided as a Supplementary File S1.

### 2.1. Research Question

The review was guided by the following research question: “What is the effect of plant-based protein supplementation on recovery from resistance exercise-induced muscle damage in healthy young adults, with specific consideration for vegan athletes?”.

### 2.2. Eligibility Criteria

The eligibility criteria for study inclusion were defined using the Population, Intervention, Comparison, Outcomes, and Study Type (PICOS) framework.

Population: Healthy young adults aged 18–44 years engaged in resistance training, including recreationally active individuals, resistance-trained individuals, and vegan athletes.

Intervention: Acute or chronic supplementation with quantified doses of plant-based proteins (e.g., soy, pea, rice, hemp, cocoa, or blends), consumed before, during, or after resistance training.

Comparison: Animal-based proteins (e.g., whey, casein), placebo/sham interventions, or no supplementation.

Outcomes: Primary outcomes included muscle recovery indicators such as DOMS, MPS, inflammatory biomarkers (e.g., CK, IL-6), and fatigue. Inflammatory markers such as exercise induced cytokines are key mediators for the delayed recovery and muscle damage. Secondary outcomes: Muscle function measures including strength, power, jump performance, and body composition indicators such as body mass index (BMI), which were interpreted in conjunction with other metrics (e.g., lean mass, fat mass) to contextualize changes.

Study Design: Randomized controlled trials (RCTs), crossover studies, and non-randomized trials published in peer-reviewed English-language journals. We have included non-randomized trials to ensure comprehensive coverage of the available evidence, particularly in areas where RCTs are limited allowing for a broader understanding of the current research landscape and supports the identification of emerging trends and gaps in the literature. Further recent empirical analysis on systematic reviews concluded that inclusion of non-randomized studies lead 89% of systematic reviews to gain statistical significance [17].

### 2.3. Information Sources and Search Strategy

A systematic search was performed across seven electronic databases: Cochrane Central Register of Controlled Trials (till 1 May 2025), Scopus (till 1 May 2025), Web of Science (till 1 May 2025), Ovid MEDLINE (1946–1 May 2025), PubMed (till 1 May 2025), ProQuest (till 1 May 2025), Cumulative Index for Nursing and Allied Health Library (till 1 May 2025), and Embase (till 1 May 2025). The search strategy was developed by an expert team under the leadership of primary author (K.G.) to ensure comprehensive coverage of the relevant literature. Key search terms and Boolean operators were utilized to capture the core concepts: plant-based proteins, resistance exercise, muscle damage, and recovery. An example search string for PubMed is: (“plant-based protein” OR “vegan protein” OR “soy protein” OR “pea protein” OR “rice protein” OR “plant protein blend”) AND (“resistance exercise” OR “resistance training” OR “strength training” OR “weight training”) AND (“muscle damage” OR “exercise-induced muscle damage” OR “delayed onset muscle soreness” OR “DOMS” OR “muscle recovery” OR “MPS”) AND (“young adults” OR “healthy adults” OR “athletes”). The search strategy administered in the other databases are displayed in Supplementary File S2. The search terms and strategy were developed as per the guidelines of PRISMA Statement for Reporting Literature Searches in Systematic Reviews [16].

The search was restricted to English language publications, with no restriction on the publication date to capture all relevant studies. Additionally, grey literature sources, including Google Scholar and backward and forward citations, were searched to identify unpublished studies. Reference lists of included studies and relevant review articles were also hand-searched to ensure comprehensive coverage. The search was conducted on 1 May 2025, with results exported to EndNote Online, https://www.myendnoteweb.com/EndNoteWeb.html) accessed on 1 May 2025, for deduplication and screening.

### 2.4. Study Selection

Two independent reviewers (K.G. and H.Z.) screened titles and abstracts of the retrieved records using Rayyan software online version. Subsequently, full texts of potentially eligible studies were assessed by the two reviewers (K.G. and K.P.) against the predefined eligibility criteria. Any discrepancies encountered during the screening or eligibility assessment phases were resolved through discussion or by consultation with a third reviewer (BK) if consensus could not be reached. The entire study selection process, including reasons for exclusion at each stage, was documented using a PRISMA flow diagram.

### 2.5. Data Extraction

The review team developed a customized data extraction form and extracted relevant information from the included studies. The data items extracted are as follows:

Study characteristics: Author(s), year of publication, country of origin, and study design. Participant characteristics: Age, sex, and training status (e.g., recreationally active, resistance-trained, vegan athletes). Resistance training characteristics: Type of exercise, intensity, duration, and specific muscle damage induction protocol. Plant-based protein characteristics: Type of protein (e.g., soy, pea, hemp), dose, timing of intake relative to exercise (pre/post), frequency, and duration of supplementation. Outcome measures: Primary outcome: Data related to DOMS, muscle function, MPS, and inflammation markers. Secondary outcome: Muscle function measures including strength, power, jump performance, and body mass index (BMI). Key findings: Principal results detailing the effect of plant-based protein on recovery outcomes compared to comparator groups.

### 2.6. Risk of Bias Assessment

The risk of bias for RCTs included in the present review were assessed using the Cochrane Risk of Bias tool (RoB 2.0). The risk of bias was assessed based on five domains: (1) randomization and allocation process (selection bias), (2) deviations from intended interventions (performance bias), (3) missing outcome data (attrition bias), (4) outcome measurement (detection bias) and (5) selection of the reported result (reporting bias). An overall risk of bias judgment was made for each outcome and each time point as either ‘low risk’, ‘some concerns’ or ‘high risk’ of bias. For non-randomized studies, if any were to be included that met the criteria, an appropriate tool such as ROBINS-I was considered. This tool assesses bias under seven domains: (1) bias due to confounding (e.g., baseline differences in training status or dietary intake), (2) bias in selection of participants (gender, team and heterogenous training and convenient sample) into the study, (3) bias in administration of protein and resistance training interventions (e.g., misclassification of protein type or dose), (4) bias due to deviations from intended interventions, (5) bias due to missing data, (6) bias in measurement of outcomes, and (7) bias in selection of the reported result. Each domain was rated as “low”, “moderate”, “serious”, “critical”, or “no information”. Two reviewers (K.G. and H.Z.) independently conducted the risk of bias assessment with disagreements resolved by discussion or a third reviewer (C.C.T.C.).

### 2.7. Data Synthesis

Given the diversity in study designs, populations (e.g., sex, training status), intervention characteristics (protein source, dose, duration, timing), and outcome measures (e.g., muscle soreness, strength, myofibrillar protein synthesis), a meta-analysis was not conducted. The included studies exhibited substantial clinical and methodological heterogeneity, which the authors decided to preclude statistical pooling by mutual consensus. As a result, a narrative synthesis was conducted. This synthesis included: Descriptive comparison of study designs, interventions, and key outcomes. Thematic grouping of studies based on protein type (single source vs. blends) and outcome domain (e.g., MPS, DOMS) and potential effectiveness on recovery after resistance training. Dose–response relationships of plant-based proteins on recovery post training.

## 3. Results

### 3.1. Study Selection and Characteristics

**Figure 1:**
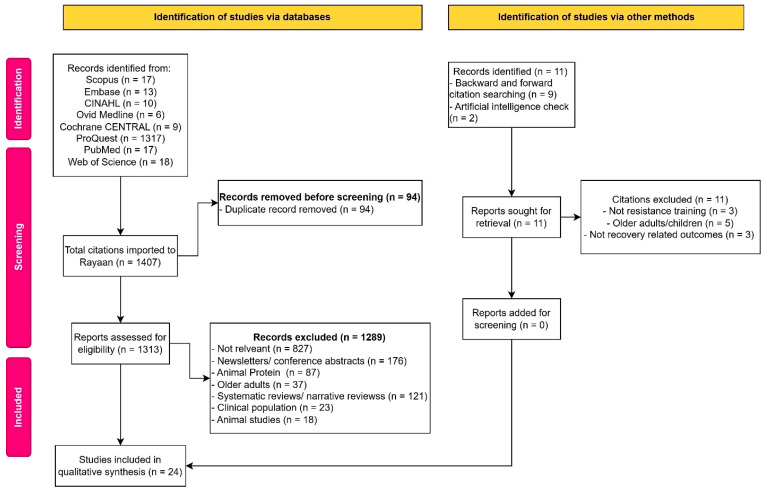
Studies screened and included in the final analysis.

The systematic search conducted on 1 May 2025, across eight databases (EMBASE, CINAHL, Scopus, Web of Science, PubMed, Cochrane Central, ProQuest and Ovid Medline) yielded 1407 studies. After deduplication, 1313 full-text articles were assessed for eligibility. A total of 24 studies were included in the final analysis based on eligibility criteria. Figure 1 shows the PRISMA flowchart that depicted the screening and inclusion of the studies for the final analysis.

### 3.2. Baseline Characteristics of the Included Studies

**Figure 2:**
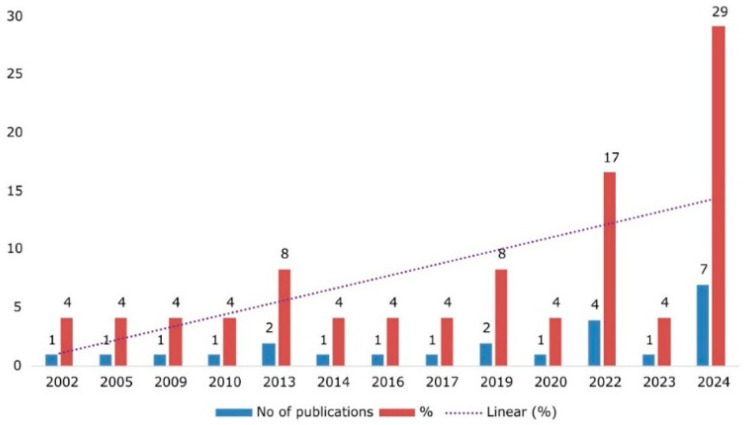
Trends in original research exploring the plant-based proteins on muscle recovery after resistance exercise training.

The majority of the studies (*n* = 23/25, 92%) were randomized controlled trials, while two studies were of non-randomized designs [5,18]. Most of the trials were assessor and participant blinded [11,19,20,21,22,23,24,25,26,27,28,29,30], while two studies employed cross over designs. These studies were conducted between 2002 and 2024 with the majority of the evidence occurred in 2024 (*n* =7/24, 29%). Figure 2 shows the publication trend that demonstrate the growing interest for plant-based protein for the recovery after resistance training.

**Figure 3:**
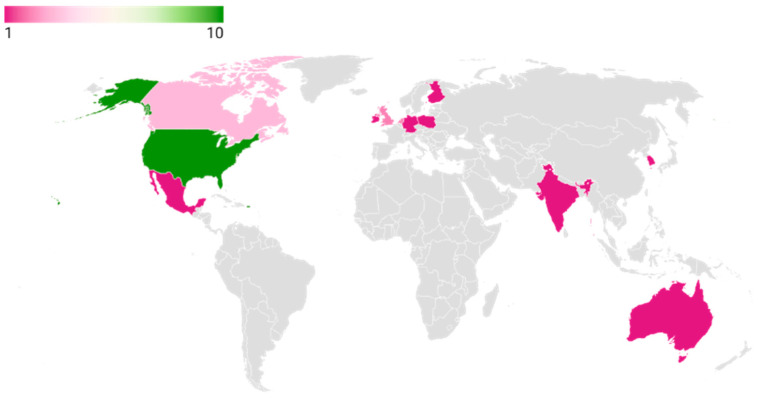
Countrywide publication trends. United States of America (*n* = 9), Canada (*n* = 3), Netherlands (*n* = 2), United Kingdom (*n* = 2), Mexico (*n* = 1), Australia (*n* = 1), Ireland (*n* = 1), Poland (*n* = 1), Finland (*n* = 1), Germany (*n* = 1), India (*n* = 1), South Korea (*n* = 1).

Almost all the studies originated from Western countries (the United States, Canada, Europe, and the United Kingdom), with only one study conducted in India [29]. Figure 3 shows the country wise publication trends.

Table 1 depicts the study demographics, participants, intervention, outcomes and the key findings.

### 3.3. Participant Characteristics

The data of 938 participants from 24 studies were included for the analysis. Most studies have administered interventions in healthy young to middle-aged adults (mean age: 18–55 years) [31], encompassing both recreationally active individuals [18,19,21,26,27,28] and those engaged in resistance-training [5,11,22,23,24,33,35]. However, research focusing on plant-based proteins in vegan athletes [31] and sedentary or inactive individuals [20,25,34] remains limited. While the majority of studies have evaluated the efficacy of plant-based proteins across mixed-gender cohorts, only a few have specifically investigated outcomes in men [5,20,22,24,25,26,27,28,35] and women [18,19] separately.

### 3.4. Intervention and Outcome Characteristics

The majority of the studies have explored Soy protein [19,20,28,29,35,36], while few studies have explored other plant-based proteins such as pea [25,27,30,34], potato [26], rice [22,24], bean [21,31], cocoa [32], hemp [23], or protein blends [11]. Few studies do not have specific plant-based protein but list a “vegan diet” [5,18,33]. Doses ranged from 15 to 40 g/day, often administered post-exercise. Control groups received whey protein, placebo, or no supplementation. The majority of the studies have explored acute effects (single session to 7 days post-exercise) [5,11,21,23,26,27,28,30,32,34,35], while few studies explored chronic effects (2–12 weeks) [18,20,22,24,29,31,33]. The majority of the studies employed supervised traditional resistance training with dose adequately elaborated [5,11,19,20,21,22,23,24,26,27,28,32,33,35] while few studies administered body weight resistance programs or not explicitly stated the training programs [18,29,31]. The primary measures often observed in most of the studies were MPS, DOMS, muscle function (isometric strength, jump performance), inflammatory markers (CK, IL-6), and recovery perceptions, while secondary measures were biomarkers of oxidative stress, amino acid bioavailability, and muscle morphology. The majority of the studies measured the muscle recovery indirectly with few measuring objectively using post-exercise MPS [11,26,27,30,35], biomarkers (CK, myoglobin and lactate dehydrogenase) [19,25,29], skeletal muscle satellite number [20], muscle thickness [23], amino acid transport rates, phenylalanine balance, and transporter expression [28]), while few measuring subjectively using self-reported perception of soreness, fatigue and readiness to train [22,29,34].

### 3.5. Effectiveness of Plant-Based Proteins on Muscle Recovery

Out of the 24 included studies, 9 reported positive effects of plant-based proteins on muscle recovery outcomes such as improved muscle protein synthesis, reduced muscle soreness, or enhanced strength recovery. These effects were more commonly observed in studies using blended plant protein formulations or higher doses (≥30 g with ~2.5 g leucine).

While the majority of studies concluded plant-based proteins including soy, potato, pea and cocoa offered no potential benefits including MPS, hormonal balance and biochemical indices [18,20,21,26,27,33,35] compared to whey, dairy or animal-based proteins, few studies concluded the positive effects of plant-based proteins on muscle recovery or fatigue perception when comparing to animal-based proteins [19,32]. While plant-based proteins have been associated with improvements in body mass index and muscle strength [22], their effectiveness in enhancing lean mass remains inconclusive [20,31]. Soy proteins did not offer upper hand in sex hormones responsible for muscle recovery during resistance training compared to whey proteins [24]. Similarly, pea proteins also were demonstrated to offer favorable effects on muscle recovery biomarkers than animal proteins [25,34], however its long-term adaptations remain uncertain [30]. Few studies demonstrated that soy proteins improved amino acid transporter proteins and offered positive phenyl alanine balance that should eventually leading to positive muscle recovery [28,29]. Kaviani et al. (2024) found gender differences with hemp on muscle recovery with females exhibiting muscle hypertrophy while males demonstrating fatigue resistance with hemp [23]. However, no significant difference in the muscle adaptations, lean body mass, strength outcomes, bone resorption and inflammatory markers [23].

### 3.6. Dose–Response Relationship of Plant-Based Proteins on Muscle Recovery

Despite the popularity of plant-based diets among resistance-trained individuals and athletes, most included studies suggest that single-source plant proteins such as soy, pea, and potato, do not possess superior benefits to animal-based proteins—particularly whey, for muscle recovery post-exercise. Studies by Tang et al. (2009) [35], Pinckaers et al. (2022, 2024) [26,27] reported that while plant proteins can stimulate MPS, their effects were often inferior or equivalent to whey, especially when leucine content or essential amino acid availability was suboptimal. However, evidence also reveals that when plant proteins are consumed in adequate doses typically in the range of 25–40 g per serving, and particularly when leucine content exceeds ≈ 2.5 g per serving, they can yield comparable outcomes to animal proteins in supporting muscle recovery [11,22,24]. For instance, Van der Heijden et al. (2024) [11] demonstrated that a plant protein blend (pea, rice, canola) matched whey in stimulating MPS rates over a 4 h post-exercise window, despite 44% lower plasma essential amino acid availability. Similarly, Joy et al. (2013) [22] and Moon et al. (2020) [24] found no significant differences in strength, lean mass, or performance gains between rice or pea proteins and whey protein when consumed post-exercise over 8 weeks. Notably, Wilkinson et al. (2023) [30] and Nieman et al. (2020) [25] found that pea protein, although beneficial in maintaining MPS, did not significantly improve recovery outcomes like DOMS or strength restoration compared to whey or placebo, indicating that acute recovery from eccentric exercise may require higher doses or multi-source formulations. Furthermore, Shenoy et al. (2016) [29] showed that soy protein, when administered at 50 g/day in trained athletes, significantly attenuated muscle damage biomarkers and improved subjective recovery, supporting a potential dose-dependent effect. While plant protein blends demonstrate promising outcomes, studies using isolated sources often show limited improvements in fatigue resistance, muscle soreness, or inflammatory biomarkers, especially in short-term interventions (e.g., 48–72 h) [37,38]. This suggests that protein quality, defined by amino acid profile, digestibility, and leucine content, plays a crucial role in the efficacy of plant proteins in muscle recovery [39]. Blended formulations (e.g., soy–dairy or pea–rice–canola) appear to overcome the amino acid limitations inherent in single-source options [40].

### 3.7. Risk of Bias Assessment

**Figure 4:**
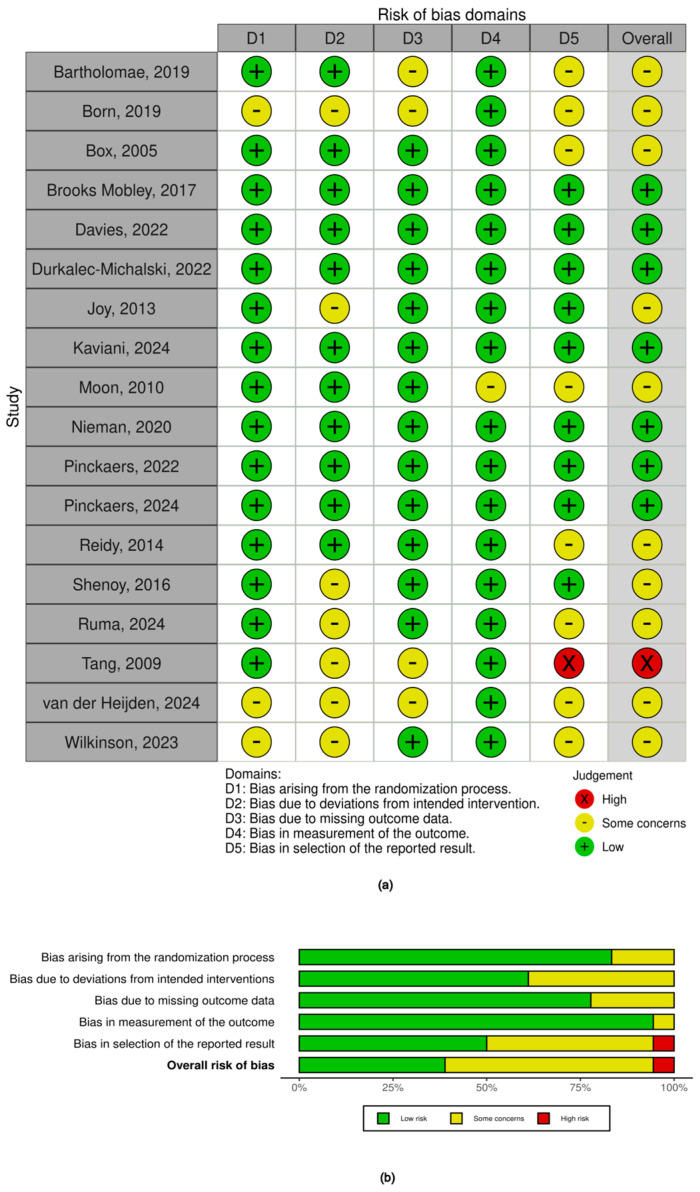
Risk of bias of randomized controlled trials (RCTs) included in the review. (**a**) showing the risk of bias of individual studies; (**b**) showing the summary of risk of bias in the RCT studies [11,19,20,21,22,23,24,25,26,27,28,29,30,31,32,33,34,35].

**Figure 5:**
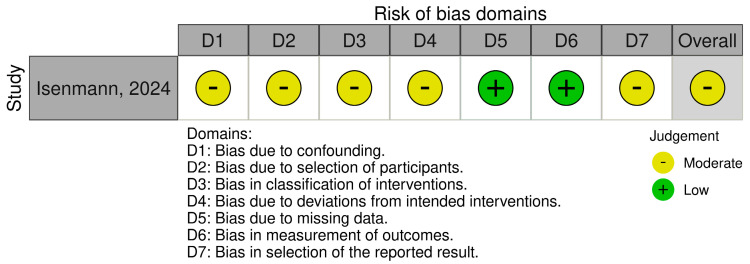
Risk of bias of non-randomized trial included in the review. Only study by Isenmann, 2024 was included for the analysis [18].

A total of 22 randomized controlled trials (RCTs) were assessed using the Revised Cochrane Risk-of-Bias Tool (RoB 2) (Figure 4a,b), while two non-randomized studies were evaluated using the ROBINS-I tool (Figure 5). Out of the 22 RCTs, 17 trials (77%) reported low risk of bias regarding the randomization process (Figure 4a). However, five trials (32%) either lacked sufficient information or had concerns due to unclear allocation procedures or baseline imbalances, indicating potential risk of selection bias [11,30,32]. In the two non-randomized studies, confounding factors were moderately controlled (Figure 5). One study adequately adjusted for key confounders such as training status and dietary intake [18], while the other study showed serious risk due to lack of statistical control for co-interventions and baseline differences. Overall, confounding was a key source of bias in the non-randomized evidence [18]. Most trials (*n* = 20) reported complete outcome data, with drop-out rates under 10% and balanced between groups. Outcome assessors were blinded in only 10 studies (45%), and self-reported outcomes such as delayed-onset muscle soreness (DOMS) and fatigue were often used without validation. As a result, detection bias was rated as some concerns or high in 12 RCTs, particularly those measuring subjective endpoints. Selective reporting of outcomes (e.g., omitting adverse effects or non-significant secondary outcomes) was a suspected ‘high risk’ in two studies [35], while ‘unclear risk’ in 12 studies (Figure 4a). Overall, the risk of bias across included studies was moderate, with several domains raising concerns (Figure 4b and Figure 5). Among randomized controlled trials, while the randomization procedures were generally adequate, detection bias and reporting bias were common due to limited blinding (Figure 4b). Among 22 randomized controlled trials, only few (*n* = 7, 32%) were classified as ‘low risk’, half (*n* = 12, 55%) fell under ‘some concerns’ or ‘moderate risk’ and two were ‘high risk’. Missing data and incomplete outcome reporting were infrequent but present in a minority of studies. For non-randomized studies, confounding (diet self-reported, sleep and other lifestyle factors influencing hormonal balance) and selection bias remained critical limitations (Figure 5). These methodological concerns should be considered when interpreting the effectiveness of plant-based proteins for muscle recovery.

## 4. Discussion

Overall, current evidence remains inconclusive regarding the efficacy of individual plant-based proteins isolates as direct alternatives to animal-based proteins for muscle recovery [22,25,26,27,30,34]. However, well-formulated plant protein blends such as those combining pea, rice, hemp and potato, have demonstrated the capacity to stimulate MPS at levels comparable to whey protein, particularly in acute post-exercise settings [5,11,28,30]. This was consistently observed in mechanistic studies using tracer methodologies [11]. However, single-source plant proteins, especially pea or soy in isolation, often failed to match whey in improving muscle function recovery or reducing DOMS within 48–72 h post-exercise [22,25,26,27,30,34]. Transitioning to a vegan diet led to challenges in maintaining protein intake and skeletal muscle mass despite dietary guidance, highlighting the difficulty of implementation without strict monitoring [18]. Our findings align with the recent systematic review and meta-analysis by Zhao et al. (2024), which concluded that while plant-based proteins confer greater benefits for MPS than no or minimal protein intake, they are still less effective than animal-based proteins [2]. Moreover, other recent systematic reviews have consistently shown that animal-based proteins are significantly more effective than plant-based proteins in enhancing muscle mass, strength, and physical performance [13,41,42,43].

Although there is increasing interest in plant-based proteins within the field of sports nutrition particularly for their potential to support MPS and recovery following resistance exercise [2,13,41,42,43], the current body of evidence remains nascent. Although vegan athletes were included in the eligibility criteria, only a limited number of studies specifically analyzed outcomes in vegan participants. As such, subgroup analyses comparing vegans to non-vegans were not feasible. This highlights a gap in the literature and underscores the need for future trials focusing on vegan populations. Many studies in our review are constrained by small sample sizes, moderate to high risk of bias, and substantial heterogeneity in the types, sources, and formulations of plant-based proteins examined [11,19,24,30,31,32,34,35]. The above limitation concurs with the recent systematic reviews [2,13,41,42,43]. These limitations hinder the ability to draw generalizable conclusions or establish definitive guidelines for their use in athletic settings. Despite these constraints, our systematic review yields several practical implications for sporting populations: (1) plant-based protein blends appear more effective than single-source plant proteins in promoting MPS and may support functional recovery; (2) adequate dosing typically 30 to 40 g per serving with approximately 2.5 to 3 g of leucine, is critical to achieving anabolic effects comparable to whey protein; and (3) nutrient timing remains essential, with immediate post-exercise intake offering potential benefits for acute recovery.

The present review findings may help athletes achieve recovery outcomes comparable to those consuming animal-based proteins, especially when using well-formulated plant protein blends [2,44]. These insights can guide coaches in designing effective post-exercise nutrition strategies. Additionally, nutritionists can use this evidence to tailor plant-based dietary plans that meet protein quality and recovery needs in resistance-trained individuals [45].

From the studies, it can be inferred that while plant-based proteins are generally safe and effective across all life stages, certain nutrients may require special attention to avoid deficiencies. These include vitamin B12, iron, calcium, vitamin D, zinc, iodine, and omega-3 fatty acids, which are either less bioavailable or present in lower amounts in plant-based sources. Athletes, especially those on vegan diets, should be advised to consume fortified foods or supplements to meet these needs. With appropriate planning and guidance, plant-based protein intake can be both nutritionally adequate and safe for supporting muscle recovery and overall health.

The potential strengths of the included studies are diverse study designs, including both acute and chronic interventions, use of objective biomarkers (e.g., CK, MPS rates) in several high-quality RCTs, comparison with gold-standard animal protein (whey) in many trials and dose–response trials in five studies clarified the role of leucine threshold (~2.5 g) for plant protein efficacy [5,20,30,35]. The general limitations of the studies included are: (1) small sample sizes (median *n* = 24), affecting statistical power; (2) heterogeneity in exercise protocols, recovery timelines, and protein formulations; (3) the quality and quantity of outcome measures varied across studies, with many relying on indirect or surrogate markers of muscle recovery which might limit the interpretation of the findings of the present review; (4) a substantial number of studies used self-reported measures such as perceived soreness and fatigue, which may introduce subjective bias; (5) only one trial [31] was conducted exclusively in vegan athletes, reducing external validity for that target population. These factors collectively limit the strength of the conclusions and highlight the need for more rigorously designed trials with standardized outcome measures and controlled nutritional protocols.

The recommendations for future research are: (1) vegan-specific RCTs: future studies should investigate plant proteins in habitual vegan athletes to assess real-world effectiveness; (2) the need of chronic trials, to evaluate long-term outcomes like muscle hypertrophy, performance, and injury recovery; (3) to examine the novel protein sources (e.g., fava bean, mung bean, algae) and fermented or hydrolyzed proteins for improved digestibility; (4) to analyze outcomes by sex, considering hormonal differences in protein metabolism, resistance training and digestibility; (5) to explore the effects of plant-based proteins in real-world contexts, future research should focus on whole-food sources and meal-based interventions, as the majority of included studies examined isolated protein supplements, limiting real-world applicability.

## 5. Conclusions

This systematic review highlights the potential of plant-based protein blends to support muscle recovery in young adults’ post-resistance exercise, as evidenced by equivalent MPS stimulation to whey in acute settings. However, single-source plant proteins like pea may not enhance functional recovery or reduce DOMS, possibly due to suboptimal leucine content or study design limitations. For vegan athletes, these findings underscore the importance of using protein blends and higher doses to meet recovery needs. Future research should focus on long-term interventions, vegan-specific populations, and optimized plant protein formulations to provide robust guidance for athletes relying on plant-based diets. By addressing these gaps, the sports nutrition field can better support the growing population of vegan athletes striving to optimize performance and recovery.

## Figures and Tables

**Table 1:** Study characteristics and findings.

Author	Year	Country	Study Design	Participants	Sample Size	Plant Protein Type	Frequency	Resistance Training Dose (C/A)	Muscle Recovery	Fatigue Outcome	Primary Outcome	Secondary Outcome	Key Findings
Bartholomae [31]	2019	United States	RCT	Healthy Less active Vegetarian adults Men and women aged 18–55	37	Mung bean protein supplement	Eighteen grams/day for 8 weeks	No structured RT program (C)	Not directly measured	Not directly measured	No primary Outcome	Changes in muscular strength (handgrip strength, knee flexor and extensor strength)	↑ in grip strength, knee flexor, and knee extensor strength in the mung protein group (+2.9% vs. −2.6%, *p* = 0.05) ↔ lean body mass between groups
Born [32]	2019	United States	RCT	High school athletes Men and women	103	Chocolate milk (CM) vs. carbohydrate (CHO)	Immediately post-exercise, 4 days per week during summer training	RT-5 weeks Bench press and squat exercises (C)	Not directly measured	Not assessed	No primary Outcome	Composite strength score (bench press + squat) individual strength measures, body weight	↑ composite strength score over time CM group had significantly greater improvements (12.3% ↑) compared to CHO group (2.7% ↑) CM led to ↑ recovery and muscle strength
Box [19]	2005	United States	RCT	Recreationally trained Young adult women	18	Soy protein isolate (Supro^®^ Soy Isolated Soy Protein)	Forty grams/day for 4 weeks	Supervised RT Three sets per exercise: bench press, lateral pull downs, military press, leg press Fewer than 3 sessions/week (C)	Indirectly assessed via creatine kinase levels	Not directly measured	Serum lipid peroxide concentrations (oxidative stress marker)	No secondary outcome	Soy protein intake ↑ pre-exercise serum antioxidant capacity Soy intake inhibited post-exercise ↑ in creatine kinase activity Lipid peroxides ↓ post-exercise in the soy group but not in the whey group
Brooks Mobley [20]	2017	United States	RCT	College-aged men Previously untrained	75	Soy protein concentrate	Two servings per day (~3 g leucine per serving) for 12 weeks	Whole-body RT Three days per week for 12 weeks Progressively loaded free-weight exercises (C)	Indirectly assessed via changes in skeletal muscle satellite cell number	Not directly measured	Indirectly assessed via changes in skeletal muscle satellite cell number	No secondary outcome	↔ skeletal muscle mass or strength Soy protein did not enhance muscle hypertrophy beyond placebo Whey protein significantly ↑ satellite cell number, Training alone led to muscle hypertrophy, independent of protein type.
Davies [21]	2022	Ireland	RCT	Healthy, young, Recreationally active adults Men and women	16	Fava bean protein (*Vicia faba* L.)	Post-exercise intake of 0.33 g/kg body mass	Unilateral knee-extensor RT Six sets of 10 maximal isokinetic contractions Three-minute rest between sets (A)	Not directly measured	Not directly measured	Myofibrillar fractional synthetic rate (myoFSR)	No secondary outcome	RT increased myoFSR (*p* = 0.012) ↔ resting or post-exercise myoFSR compared to control ↔ MPS responses
Durkalec-Michalski [33]	2022	Poland	RCT	Moderately trained CrossFit practitioners Men and women	20	Vegan diet (VegD) vs. mixed diet (MixD)	Diet adherence for 4 weeks, monitored daily	Three sessions per week for 4 weeks progressive overload (60–100% 1RM) (C)	Not directly measured	Not directly measured	Blood biochemical indices (lipid profile, iron metabolism, glucose levels, liver function)	RT (squat and deadlift) 70% 1RM	↔ exercise performance between vegan and mixed diet groups ↑ deadlift repetitions in the VegD group ↑ squat repetitions in the MixD group
Goldman [5]	2024	Finland	Modeling study	Competitive male bodybuilders	235	Completely plant-based diet	Scaled to daily caloric intake of 4239 kcal	Four to seven sessions/week Session lasting 60–90 min Three to four sets/exercise Seven to twelve reps per set (C)	Not directly measured	Not measured	Protein and leucine adequacy for hypertrophy	No secondary outcome	Leucine intake for hypertrophy (≥2 g/meal, 11 g/day) ↔ no change in recovery
Isenmann [18]	2024	Germany	Non-randomized trial	Young, recreationally trained women	10	Vegan diet (no specific protein supplementation)	Eight-week vegan phase followed by 4-week omnivorous phase	Participants maintained habitual RT regimes No prescribed RT protocol (C)	Not directly measured	Not directly measured	Menstrual cycle tracking (hormonal fluctuations, cycle length)	Changes in body composition Performance assessments (squat, countermovement jump)	↑ Increase in CHO consumption during the vegan phase body weight ↓ and skeletal muscle mass ↓ during the vegan phase ↔ squat performance. ↓ countermovement jump height
Joy [22]	2013	United states	RCT	Twenty-four resistance-trained college-aged men	24	Rice protein isolate	Forty-eight grams of rice or whey protein isolate consumed post-exercise on training days for 8 weeks	Three RT sessions per week for 8 weeks Non-linear periodized training targeting major muscle groups (C)	soreness, perceived readiness to train, recovery scales)	Perceived readiness to train	Ratings of perceived recovery, soreness, and readiness to train	Changes in body composition Muscle thickness and strength	Both rice protein and whey protein ↑ lean body mass, muscle hypertrophy, strength, and power. ↔ muscle growth or performance gains.
Kaviani [23]	2024	Canada	RCT	Trained young adults Men and women	34	Hemp protein powder (40 g protein, 9 g oil per day)	Sixty grams per day, divided into two doses	Eight-week program Four sessions/week Three to four sets of 4–10 repetitions Between 75 and 90% 1RM to volitional fatigue (C)	Indirectly assessed through muscle thickness	rate of torque development after fatigue test	Inflammation markers (C-reactive protein, Interleukin-6)	Lean tissue and fat mass (DXA scanning) Muscle hypertrophy (ultrasound measurements)	↑ elbow flexor muscle thickness Hemp group preserved twitch torque and rate of torque development
Moon [24]	2020	United states	RCT	Healthy RT trained men	24	Rice protein concentrate	Twenty-four grams of rice protein concentrate daily for 8 weeks	Four workouts per week (2 upper-body, 2 lower-body sessions) Linear periodized training program Predetermined progression (C)	Not directly assessed	Not directly assessed	No primary Outcome	Changes in body composition (fat-free mass, fat mass, lean mass) muscular strength (bench press and leg press 1RM)	↔ body composition or performance outcomes. ↑ fat-free mass, lean mass, bench press 1RM, and leg press 1RM ↔ muscular endurance, anaerobic power, or fat loss between groups.
Nieman [25]	2020	United States	RCT	Non-athletic, non-obese men aged 18–55 years	92	Pea protein isolate (NUTRALYS^®^ S85 Plus)	0.9 g protein/kg per day divided into three doses for five days post-exercise	Eccentric exercise bout for 90 min RT, plyometric movements, and downhill treadmill running	biomarkers (creatine kinase, myoglobin, lactate dehydrogenase)	Not directly measured	Muscle damage biomarkers (creatine kinase, myoglobin) DOMS Inflammation markers (CRP)	Physical fitness test performance (bench press, Wingate anaerobic test, vertical jump, leg-back strength)	Whey protein ↓ post-exercise muscle damage biomarkers (creatine kinase, myoglobin) compared to water. ↔ whey and pea protein groups. ↔ muscle soreness or physical performance during recovery.
Pinckaers [26]	2022	Netherlands	RCT	Healthy, young recreationally active men	24	Potato protein concentrate (Solanic 100)	Single ingestion of 30 g of potato protein post-exercise	Unilateral RT (leg press and knee extension machines) 3 sets of 8 repetitions at ~80% 1RM, plus one set to failure (A)	Assessed through post-exercise MPS rates	Not directly measured	Mixed MPS rates at rest and during recovery from RT	No secondary outcome	↑ MPS rates ↔ MPS rates between potato and milk protein. Post-exercise MPS rates ↑ ↓ plasma amino acid availability
Pinckaers [27]	2024	Netherlands	RCT	Healthy, young recreationally active men	24	Pea protein concentrate (Nutralys S85F)	Single ingestion of 30 g of pea protein post-exercise	Unilateral RT Three sets of 8 repetitions at ~80% 1RM, plus one set to failure (A)	Assessed through post-exercise MPS rates	Not directly measured	Post-prandial MPS rates following pea vs. milk protein ingestion	No secondary outcome	Milk proteins- ↑ plasma essential amino acid concentrations pea protein ↔ milk protein in MPS rates
Reidy [28]	2014	United states	RCT	Healthy, young, recreationally active men	16	Soy-dairy protein blend (25% soy, 50% casein, 25% whey) vs. whey protein isolate	Single post-exercise ingestion (1 h after RT)	High-intensity leg RT Eight sets of 10 reps leg extension machine 55–70% 1RM (A)	Assessed via MPS (amino acid synthesis)	Not directly measured	Muscle amino acid transport Phenylalanine net balance and transport rate MPS	No secondary outcome	Soy ↔ whey ↑ amino acid transporter expression, amino acid transport into muscle, and MPS
Shenoy [29]	2016	India	RCT	Trained male athletes (20 boxers, 20 cyclists), aged 18–28 years	40	Isolated Soy Protein (ISP)	Twenty-five grams of ISP twice daily (mixed with water) for 4 weeks	One-hundred drop-jumps Five sets of 20 consecutive jumps, Ten-second intervals between jumps Two-minute rest between sets (C)	Inflammatory markers, Myeloperoxidase and Isometric muscle strength	Visual Analog Scale (VAS) for muscle soreness	Changes in biochemical markers of muscle damage, inflammation, and oxidative stress perceived muscle soreness	Isometric muscle strength, aerobic capacity (VO2 max),	Soy protein ↓ muscle damage and inflammation markers ↑ muscle recovery observed in boxers than cyclists following supplementation.
Ruma [34]	2024	Canada	RCT	Healthy, sedentary adults aged 30–59 years	50	Pea protein powder (NUTRALYS^®^ S85 Plus)	Between 20 and 22.5 g per day, mixed with water and consumed post-exercise	Six sessions per week (30 min each) Three upper-body and three lower-body sessions Exercises performed to fatigue with self-selected resistance (C)	Assessed via DOMS questionnaire at 24 h, 48 h, and 72 h post-exercise	Not directly measured	Exercise recovery (muscle soreness tracking)	Muscle strength Endurance performance via treadmill walk test Changes in body composition (DXA scanning: muscle mass, fat mass)	Pea protein ↑ 16.1% in WBMS, compared to 11.1% for whey protein Exercise recovery ↑ with pea protein, ↓ muscle soreness scores at 24 h, 48 h, and 72 h post-exercise.
Tang [35]	2009	Canada	RCT	Healthy, young men Regularly engaged in RT (2–3 days per week)	18	Soy protein isolate	Single ingestion	Unilateral leg resistance exercise Four sets of leg press and knee extension exercises at 10–12 RM intensity (A)	Evaluated through MPS measurement	Not directly measured	Rates of mixed MPS at rest and post-exercise Blood amino acid concentrations Muscle anabolism	No secondary outcome	Whey protein ↑ in MPS Post-exercise MPS was 122% ↑ with whey vs. casein and 31% ↑ whey vs. soy Whey ↑ essential amino acids and leucine
van der Heijden [11]	2024	United Kingdom and United states	RCT	Healthy, RT, young adults male/female: age: 26 ± 6 years	10	Protein blend composed of pea (39.5%), brown rice (39.5%), and canola (21.0%)	Single ingestion (32 g of protein) post-exercise	Bilateral leg RT 4 sets of safety bar squat, leg press, and leg extension 10–12 RM intensity (A)	Assessed via MPS	Not directly measured	Postexercise MPS rates Plasma amino acid concentrations	No secondary outcome	Postexercise MPS rates ↔ Whey protein ↑ essential amino acid concentrations (~44% higher than plant protein),
Wilkinson [30]	2023	United Kingdom	RCT	Healthy, recreationally active Men and women	19	Pea protein fortified with methionine	A combination of 25 g protein + 2.2 g leucine daily, post-exercise for 7 days	Three-hundred maximal eccentric contractions Ten sets × 30 reps) Four sets of 30 isokinetic knee extensions per day (A)	MPS, Soreness	Not directly measured	Muscle soreness following eccentric exercise MRI-based muscle volume	No secondary outcome	Pea protein ↔ muscle function recovery or ↓ soreness compared to placebo. MRI scans ↔ muscle swelling post-exercise suggesting soreness

Abbreviations: C—Chronic effect, A—Acute effect, BMI—body mass index, CHO—carbohydrate, CM—chocolate milk, CRP—C-reactive protein, DOMS—Delayed onset of muscle soreness, DXA—dual X-ray absorptiometry, MixD—mixed diet, myoFSR—Myofibrillar fractional synthetic rate, MPS—muscle protein synthesis, RCT—randomized controlled trial, RM—repetition maximum, RT—resistance training, VegD—vegan diet, VAS—Visual Analog Scale, VO2max—maximal oxygen consumption, WBMS—Whole-body muscle strength, ↑ increased effect, ↓ decreased effect; ↔ equivocal or no difference effect.

## Data Availability

All the included studies are in article.

## Supplementary Materials

The following supporting information can be downloaded at: https://www.mdpi.com/article/10.3390/nu17152571/s1, File S1: PRISMA (Preferred Reporting Items for Systematic Reviews and Meta-Analyses) guidelines, utilizing the PERSiST (Preferred Reporting Items for Systematic Reviews in Sport and Exercise Science) guidance checklist; File S2: Search strategies employed in different databases.

## Abbreviation

IL-6	interleukin-6
